# Comparative spatial whole transcriptome analysis of matched frozen and formalin-fixed paraffin-embedded colorectal cancer tissues

**DOI:** 10.1016/j.bbrep.2025.102413

**Published:** 2025-12-16

**Authors:** Kullanist Thanormjit, Thanawat Suwatthanarak, Tantip Arigul, Amphun Chaiboonchoe, Onchira Acharayothin, Piroon Jenjaroenpun, Vitoon Chinswangwatanakul, Pariyada Tanjak

**Affiliations:** a Siriraj Cancer Center, Faculty of Medicine Siriraj Hospital, Mahidol University, Bangkok, 10700, Thailand; b Department of Surgery, Faculty of Medicine Siriraj Hospital, Mahidol University, Bangkok, 10700, Thailand; c Division of Medical Bioinformatics, Research Department, Faculty of Medicine Siriraj Hospital, Mahidol University, Bangkok, 10700, Thailand; d Siriraj Long-read Lab (Si-LoL), Medical Bioinformatics Lab, Siriraj Genomics, Faculty of Medicine Siriraj Hospital, Mahidol University, Bangkok, 10700, Thailand; e Siriraj Center of Research Excellence for Systems Pharmacology, Faculty of Medicine Siriraj Hospital, Mahidol University, Bangkok, 10700, Thailand

**Keywords:** Spatial whole transcriptomics, Colorectal cancer, Formalin-fixed paraffin-embedded, Fresh frozen, Snap frozen

## Abstract

Spatial transcriptomic technology enables resolving a tumor microenvironment heterogeneity of colorectal cancer (CRC) tissue samples. However, there is limited evidence regarding the relative strengths and weaknesses of different CRC sample types and preservation methods, including formalin-fixed paraffin-embedded (FFPE), fresh frozen (FF) and snap frozen (SF). Here, we examine gene expression profiles in 36 tissue areas, FFPE, FF and SF samples, derived from a patient with stage IV CRC, using spatial analysis of the whole transcriptome. The tissue samples obtained from surgical resection of the cancer were subjected to a detailed histological evaluation and spatial transcriptomics analysis to compare the quality of the sample between different preservation methods. Spatial transcriptomic analysis demonstrated that FFPE tissue samples offered improved resolution of cellular morphology in the tumor microenvironment, facilitating greater detail of gene expression and cell types. In contrast, gene expression analysis of FF and SF tissues embedded in optimal cutting temperature compound revealed a high level of CRC gene expression. FFPE, FF and SF samples shared a significant overlap among the top 300 genes that were strongly associated with key biological processes in CRC. In summary, the spatial analysis of the whole transcriptome across FFPE, FF and SF CRC tissues revealed intratumoral heterogeneity and highlighted key transcriptomic signatures, suggesting the specific value of different tissue preservation methods in the profiling of CRC.

## Highlights

• Spatial transcriptomics applied to matched FFPE, FF, and SF CRC tissues.
• FFPE preserved spatial context; FF/SF retained higher transcript abundance.
• Shared 132 DEGs across all tissue types linked to key CRC pathways.
• FFPE enables reliable spatial profiles and preserves transcriptomic signatures.

## 1 Introduction

CRC is a heterogeneous disease characterized by diverse genetic alterations and disruptions in multiple signaling pathways, occurring both within tumor cells and the tumor microenvironment (TME), which collectively contribute to its progression and invasiveness [1,2]. Although consensus molecular subtypes of CRC have emerged [3], the heterogeneity inherent to CRC presents significant challenges for effective clinical management, emphasizing the need for precise molecular characterization of tumor tissues [4]. Recently, single cell and spatial transcriptomics was applied to CRC samples, revealing the intratumoral heterogeneity of CRC and its microenvironment in individual patients [[5], [6], [7]]. However, scRNA-seq alone provides limited insight into the spatial or anatomical context inherent in tissue architecture.

To address these limitations, spatial transcriptomic technologies aim to provide comprehensive molecular insights while preserving the histological architecture of tissues. Spatial transcriptomic technology enables to combine spatial information and gene expression profiling which has emerged as a valuable tool for unraveling the complexity of TME [1,[8], [9], [10]]. The Nanostring GeoMx Digital Spatial Profiler (DSP) is one of commercially available technologies for conducting spatial transcriptomics and proteomics that enables high-level multiplex spatial profiling of proteins and RNA in tissue samples within a defined region of interest (ROI) [11]. The basic principle of DSP relies on a morphology-guided approach using UV-photocleavable oligonucleotide barcodes conjugated to antibodies or RNA probes that hybridize to multiplex immunofluorescence–stained tissue sections [11]. Upon UV exposure, barcodes from user-defined ROI are released, collected, and subsequently quantified either by the NanoString nCounter or next-generation sequencing platforms [11]. The DSP platform is currently employed to understand the mechanisms underlying metastatic development [10,12], disease heterogeneity [13], and prognosis [14] of CRC.

The Siriraj Cancer Center, Siriraj Hospital Faculty of Medicine, Mahidol University, Thailand, established a CRC pilot biobank. Surgical specimens and peripheral blood samples from more than 500 patients with stage I–IV CRC have been collected and preserved using FFPE, FF and SF methods. We have reported several studies utilizing CRC samples from our biobank [1,9,10,[15], [16], [17], [18], [19]]. Differences in preservation methods can lead to variability in RNA integrity and gene expression profiles [[20], [21], [22]]. This variability may introduce technical biases that affect the accuracy and reproducibility of downstream analyses, including transcriptomic and spatial transcriptomic profiling [11,23]. Recognizing these potential limitations is crucial to interpret molecular data and ensure that findings derived from each CRC sample remain meaningful without losing of significant information.

In this work, we use the DSP with a whole transcriptome atlas (WTA) panel, which enabled spatially resolved analysis of gene expression in various preservation methods [11]. However, to our knowledge, no side-by-side comparisons have been made in FFPE, FF and SF clinical tissue samples from the same patient. To investigate which features of CRC tissue are preserved in archival samples, we performed a side-by-side profiling of CRC tissues preserved as FFPE, FF, and SF specimens. This study aims to serve as a proof-of-concept feasibility investigation evaluating the technical comparability of different preservation methods. Our findings demonstrate that FFPE samples maintained adequate transcript integrity and spatial resolution.

## 2 Methodology

### Ethical approval

A participant was informed about the study through both verbal explanation and written documentation as well as provided signed written informed consent prior to enrollment. The study protocol and the informed consent procedures were reviewed and approved by the Siriraj Institutional Review Board (certificates of approval number Si105/2021). All methods were performed in accordance with the relevant guidelines and regulations.

### 2.1 Tissues from a patient with CRC

Surgical specimens were collected from a patient with CRC at Siriraj Hospital and all experiments were conducted during February 6th, 2021–February 4th, 2024. The tissues were removed in the course of routine surgical procedures according to diagnostic requirements in 2021. Spatial whole transcriptome analysis was conducted and analyzed in 2024. The storage time of all specimens were approximately 3 years. For FF, the tissue was promptly cut into 2–3 mm pieces within 30 min after surgical resection, then immersed in 1 ml of chilled RNAlater solution (Invitrogen, MA, USA), following the protocol outlined in our previous report [15,19]. The FF samples were stored at −80 °C in the Siriraj CRC biobank. For SF, the tissue was rapidly frozen in liquid nitrogen immediately after collection.

For FFPE, tissue was fixed in formalin and embedded in paraffin, the standard method for long-term storage at room temperature [19]. In brief, the resected primary CRC tissues were fixed in 10 % neutral buffered formalin within 60 min after surgical resection. The fixation process lasted for three days. Subsequently, the fixed tissues were dehydrated through ascending grades of ethanol and cleared by immersion in xylene. For infiltration, the dehydrated and cleared tissues were immersed in molten paraffin wax. After infiltration, the tissues were oriented in molds filled with molten paraffin wax and allowed to solidify on a cold plate set at −5 °C. Finally, the resulting paraffin blocks containing the embedded tissue were stored at ambient temperature.

### 2.2 Tissue preparation for spatial whole transcriptome analysis

CRC tissues, FFPE, FF and SF, were extracted and evaluated for the RNA integrity by distributive value 200 (DV200). All tissues had DV200 > 50 %. Slide tissue preparation employed standard protocols of the GeoMx DSP slide preparation (MAN-10150-03/2023, NanoString Technologies, WA, USA). Briefly, FF and SF tissues were mounted onto cryostat chucks using optimal cutting temperature (OCT) compound (Sakura Finetek, CA, USA). The OCT was frozen at cryosectioning temperature for 5 min. The OCT tissues were cut at 5 μm and mounted on Superfrost Plus slides (VWR International, PA, USA) using a cryotome (Leica CM1950, Nussloch, Germany). After mounting, the slides were placed on dry ice for 1 h. For FFPE, tissue block was cut at 5 μm and mounted on Superfrost Plus slides (VWR International, PA, USA) using microtome (Leica RM2235, Nussloch, Germany).

### 2.3 The GeoMx DSP

All slides were performed according to the manufacturer's user manuals (MAN-10150, MAN-10152, MAN-10153, and MAN-10154, NanoString Technologies, WA, USA) and as previously described [9,11]. A tricolor panel of fluorescence morphology markers (NanoString Technologies, WA, USA) was used to label Pan-cytokeratin (PanCK; epithelial and tumor cells), CD45 (immune cells), and SYTO13 (nuclei) on the stained slides. The slides were loaded onto the GeoMx® instrument, scanned, and selected for the ROI (n = 12). Each ROI was UV-illuminated three times, once for the PanCK+ segment, once for the CD45^+^ segment, and once for the PanCK-/CD45- segment. The resulting areas of illumination (AOIs, n = 36) had a surface area in the range of 2477 - 209,138 μm^2^, encompassing between 52 and 2181 nuclei.

Initially, we selected 12 regions of interest (ROIs) of the same size. However, each ROI was subsequently UV-illuminated three times to generate three AOIs, which resulted in AOIs with different areas and numbers of nuclei that reflected the biological heterogeneity of the tissue. The rationale for using variable-sized areas in this study is that it allows a more accurate comparison of transcriptional differences among distinct regions in each sample type. Following ROI selection, photocleavable oligonucleotides (barcodes) for individual targets were UV-cleaved, collected and quantified using next-generation sequencing (Illumina NovaSeq 6000, illumina, CA, USA) by sending libraries preparation to Novogene Co., Ltd. (Singapore). The generated raw FASTQ data was subsequently demultiplexed according to the DSP system to generate a count matrix for downstream analysis.

### 2.4 Data analysis

Raw probe level counts were subjected to biological probe quality control (QC) and normalization following NanoString's Human WTA Normalization guidelines (MAN-10154, NanoString Technologies, Inc., WA, USA). AOI QC was performed in the DSP Analysis Suite (software version 3.1.0.194). Biological probe QC was performed with the default settings excluding probes from the target count calculation in all segments if the geomean probe in all segments/geomean probes was within the target ≤0.1 and if the Grubbs' outlier test failed in ≥20 % of the segments. If a probe failed the Grubbs' outlier test in a specific segment, the probe was excluded from the target count calculation in that segment. The limit of quantitation (LOQ) was calculated using two standard deviations of the negative probes.

### 2.5 Normalization

Three normalization methods were utilized. Quality control and filtering were conducted according to the manufacturer's protocol (MAN-10154-04, GeoMx DSP software version 3.1.0.194). Both methods were performed in the DSP analysis suite. First, after LOQ calculation, expression filtering was performed, which kept all targets that are above LOQ with 10 % of AOIs. Quartile 3 count (Q3) normalization was performed after filtering the dataset to remove targets below the LOQ. Second, Q3 was performed without filtering the expression. Third, the trimmed mean of M-values (TMM) was calculated using the Bioconductor R package standR (Spatial transcriptomics analyzes and decoding in R) [24]. An ANOVA test was used to assess differences among the three normalization methods. The comparison was based on the average expression levels across all genes for each method. Statistical significance was established at p-value <0.05.

### 2.6 Differential expressed gene (DEG)

The Q3-normalized counts were subsequently processed using the DESeq2 R package to examine expression profiles through Principal Component Analysis (PCA) [25]. DEG analyzes were performed separately for FFPE, FF and SF to compare the expression between PanCK-/CD45- and other segments (PanCK+ and CD45+) within each group. The Q3-normalized data without 10 % filtering expression, together with log2 fold changes and the Wald test-adjusted p-value (p-adj <0.05), were extracted using the results function from DESeq2 [26].

### 2.7 Functional enrichment of DSP data

To compare differential gene expression profiles across the three tissue types, we selected the top 300 upregulated genes from each group. Pairwise comparisons of the log10-transformed expression values for these genes were performed using the ggpairs function from the GGally R package (https://ggobi.github.io/ggally, https://github.com/ggobi/ggally) with Pearson's correlation used for statistical testing (p-value <0.001).

Intersecting genes among the three tissues were identified using the ggvenn package. These overlapping genes were subjected to gene ontology analysis using the org.Hs.eg.db package in R, applying the Benjamini-Hochberg correction with a significance threshold of p-adj <0.05. KEGG pathway enrichment analysis was also performed using a relaxed threshold of p-adj <0.05 to identify pathways associated with cancer.

## 3 Results

**Fig. 1:**
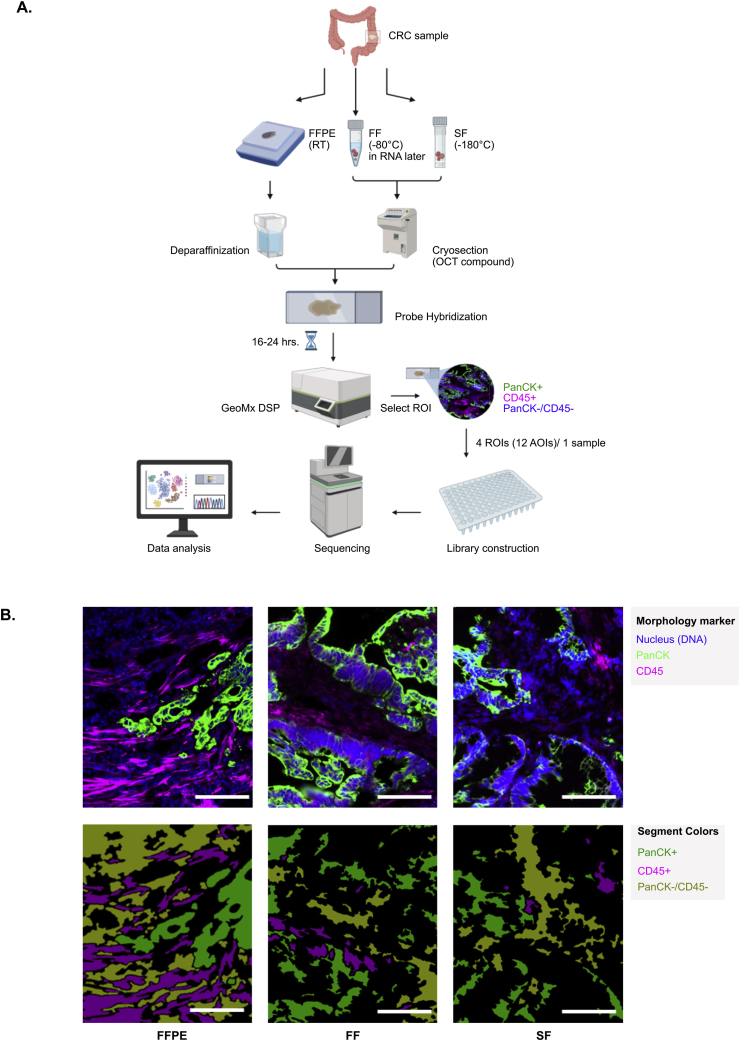
**Schematic diagram of the experimental study. (**A) A primary colorectal cancer (CRC) from a patient with stage IV CRC was collected and preserved using fixed-formalin paraffin-embedded (FFPE), fresh frozen (FF) and snap frozen (SF) methods. Whole transcriptomic profiles of all tissues were evaluated by using the GeoMx DSP platform. The illustration was created in BioRender.com/m71p068. (B) Representative image of PanCK+, CD45+, and PanCK-/CD45- segments detected by the GeoMx instrument; scale bars, 100 μm.

We studied the potential of an FFPE CRC sample in spatial whole transcriptome analysis using the GeoMx DSP. We used a tricolor fluorescence morphology panel—PanCK to identify epithelial and tumor cells, CD45 to mark immune cells, and SYTO13 for nuclei staining—to guide the selection of ROIs. Whole transcriptome data obtained from ROIs in FFPE tissue were compared with matched FF and SF tissues, which are samples from the primary CRC of the same patient as shown in Fig. 1A. To enhance statistical power, we select four ROIs in each tissue. In a total of 12 ROIs with triple segments including PanCK+, CD45+, and PanCK-/CD45- (36 AOIs), the representative fluorescence and segmented images are shown in Fig. 1B.

### 3.1 Normalization data

**Fig. 2:**
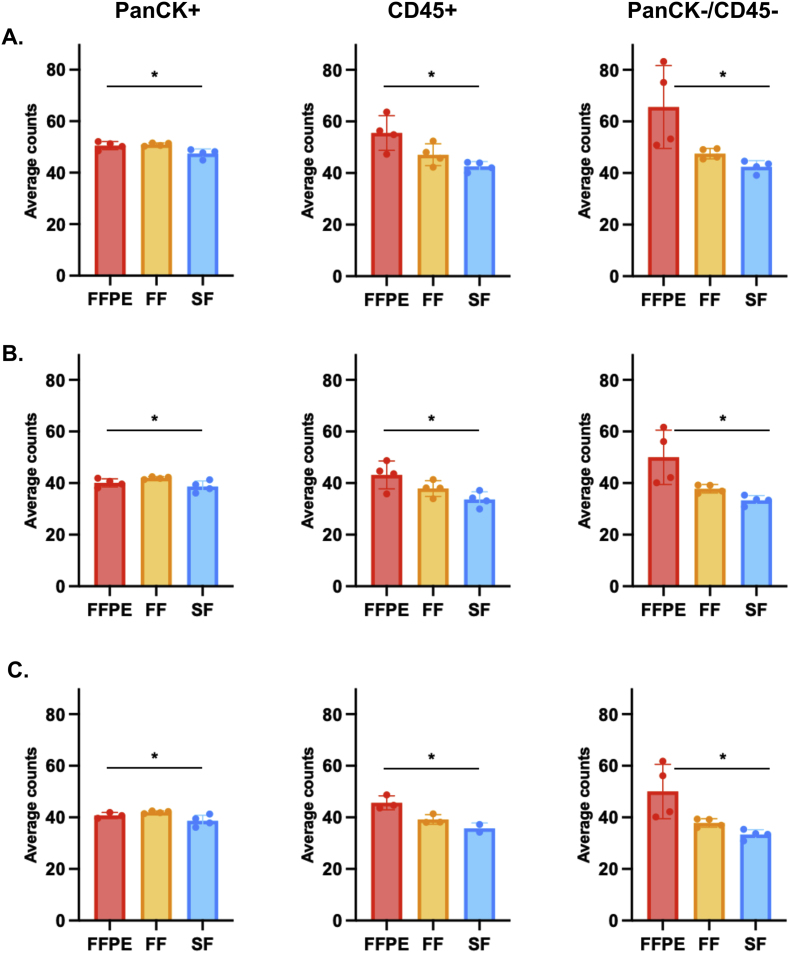
**Normalization data from three different methods.** (A) Q3 normalization was performed after filtering the dataset to remove targets below the LOQ with 10 %. (B) Q3 was performed without filtering expression. (C) TMM. PanCK+ (left), CD45+ (middle) and PanCK-/CD45- (right). An ANOVA test was used to determine whether the three normalization methods differed by comparing the average expression levels across all genes for each normalization method (∗, p-value <0.05).

To ensure that all segments had similar gene expression ranges and that there were no biases from the area and nuclei, we initially employed three different methods to normalize the raw counts from the GeoMx platform. The average expression level across all genes obtained from different normalization methods for each segment in FFPE, FF and SF tissues are presented in Fig. 2A–C. Different normalization methods exhibited similar trends in the data, suggesting that normalization performed using either the DSP Analysis Suite (software version 3.1.0.194) or alternative analytical methods yields similar outcomes. All normalization methods in this study can reduce the variance from segment size, segment cellularity, and other technical factors.

However, the results of Q3 normalization with filtering applied to exclude gene expression levels below the 10 % LOQ (Fig. 2A) demonstrated higher average expression level across all genes than the results of Q3 without filtering (Fig. 2B) and TMM (Fig. 2C). Interestingly, CD45+ and PanCK-/CD45- segments in FFPE tissue showed the highest average expression level across all genes, suggesting that utilizing FFPE tissue in spatial analysis will be essential to elucidate the architectural complexity of the TME in CRC tumors. Then, we used Q3 normalization without expression filtering for all subsequent downstream analyzes.

### 3.2 Spatial whole transcriptomic profiles reveal inter-tissue and intra-tissue heterogeneity of CRC tumor

**Fig. 3:**
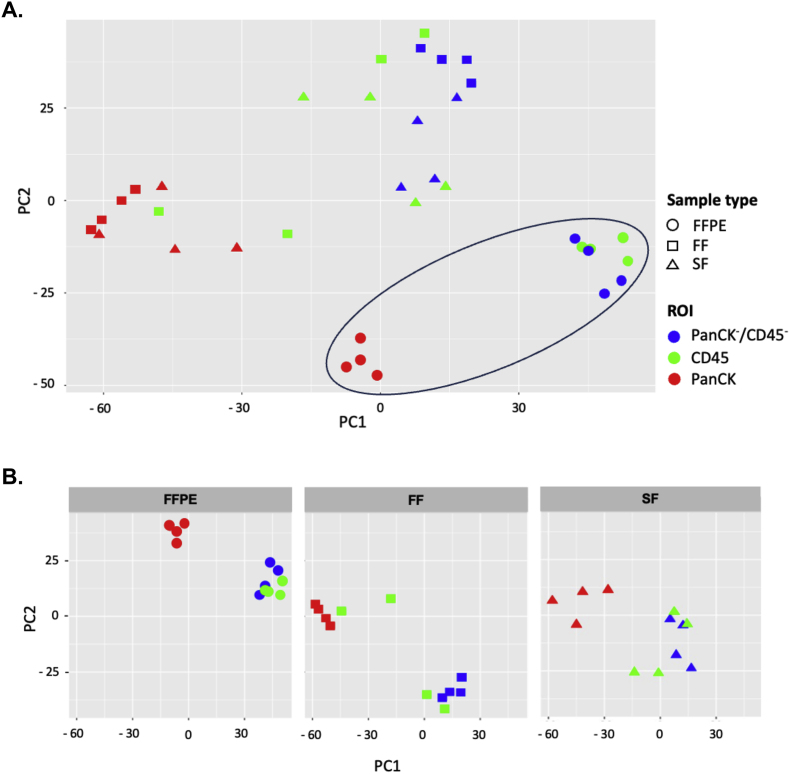
**The heterogeneity among tissue types**. (A) PCA embeddings of the gene expression measurements in segment-annotated spots which were colored PanCK+ (red), CD45+ (green), and PanCK-/CD45- (blue) of FFPE (circle), FF (square), and SF (triangle). (B) PCA embeddings of the gene expression measurements in segment-annotated spots which were colored PanCK+ (red), CD45+ (green), and PanCK-/CD45- (blue) of each tissue type, FFPE (left), FF (middle), and SF (right).

To investigate the heterogeneity among tissue types, PCA embeddings of the gene expression measurements were performed. We found that the gene expression measurement in segment-annotated spots of FF and SF were distinct from those of FFPE (Fig. 3A). We further characterized the intra-heterogeneity of segments-annotated spots across the different tissue preservation methods (Fig. 3B). The results revealed that PanCK + segment-annotated spots of FFPE and SF tissues were clustered and clearly separated from CD45+ and PanCK-/CD45- segment-annotated spots. In contrast, for FF tissues, a subset of CD45+ segment-annotated spots clustered within the PanCK+ segment.

This observation may suggest both the mixed cellular composition of the tissue and limitations of the segmentation approach. Because each ROI, which contains a mixture of cell types, was UV-illuminated three times to generate three AOIs, some CD45+ segments may have captured adjacent stromal areas. As a result, the immune signal could become diluted and appear similar to the PanCK-/CD45-compartment. Conversely, some PanCK-/CD45- segments may include immune cells, making them appear more similar to CD45−segments, as the immune cell-type deconvolution between CD45−and PanCK-/CD45- segments showed similar cell-type patterns of across FFPE, FF and SF samples (Supplementary Fig. 1). To further explore these observations, we performed differential gene expression analyzes by comparing PanCK+ and CD45+ segments with PanCK-/CD45- segments.

### 3.3 DEGs and shared genes across different tissue types

**Fig. 4:**
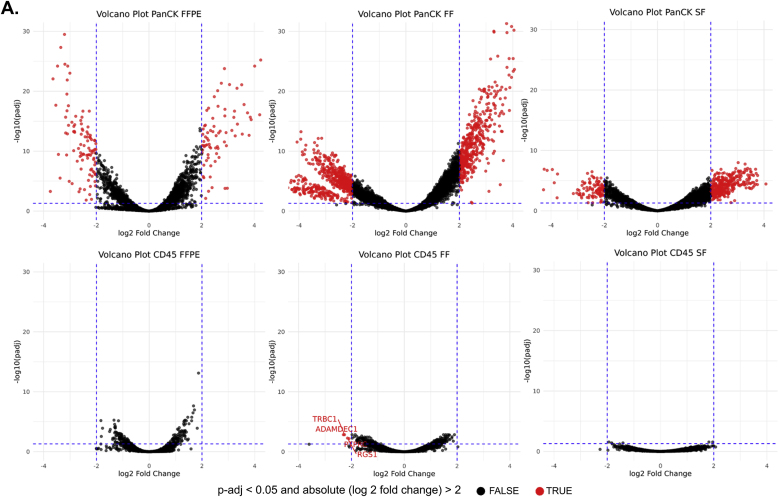

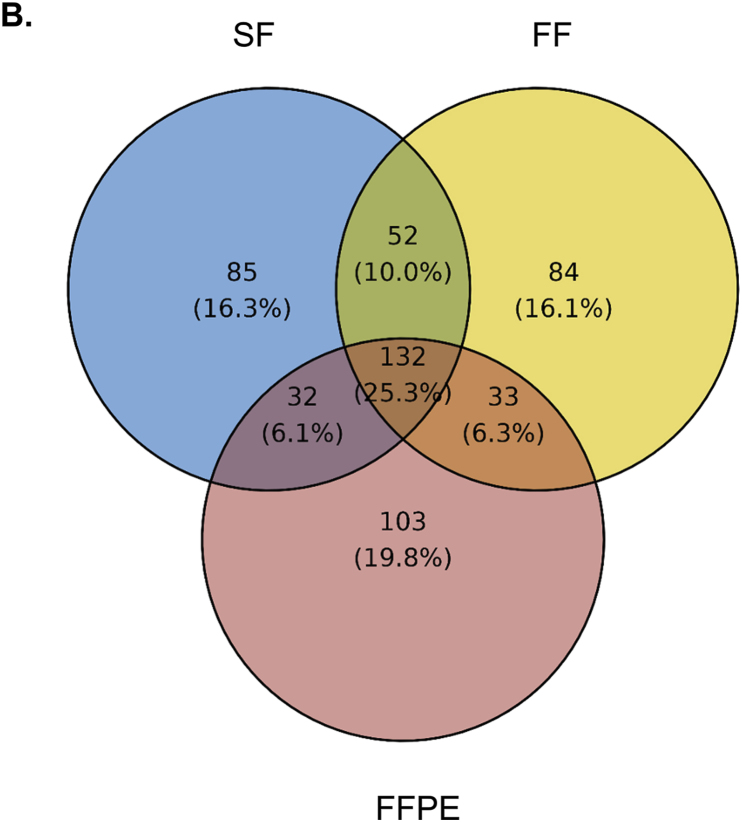

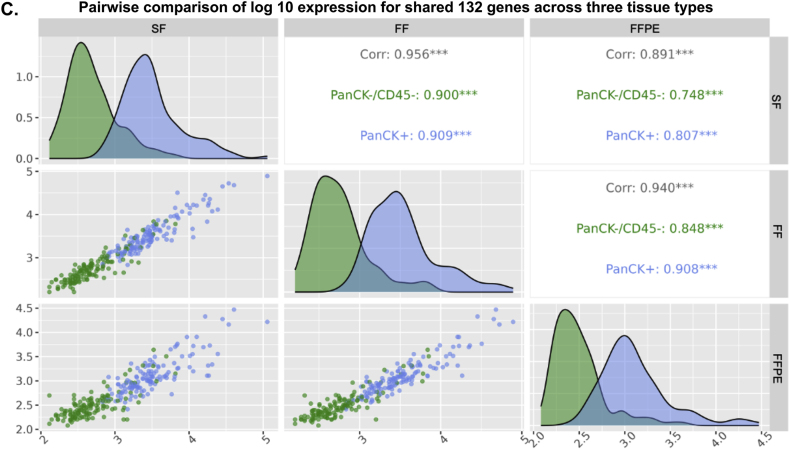
**Differential gene expression and shared gene across different tissue types.** (A) Volcano plots comparing DEGs between PanCK+ and PanCK-/CD45- segments (Top), and between CD45+ and PanCK-/CD45- segments (Bottom) for FFPE (left), FF (middle), and SF (right). Statistical significance was established at p-adj <0.05 and absolute log_2_ fold change >2 (B) Venn diagram showing the intersection of top 300 DEGs between PanCK+ and PanCK-/CD45- segments across three different sample types. (C) Density plots of the pairwise scatter plots (ggpairs) from the pairwise comparison analysis showing the distribution of 132 intersecting DEGs between PanCK+ (blue) and PanCK-/CD45- (green) segments in FFPE, FF, and SF samples. Diagonal panels display the density distributions and the upper panel report Pearson correlation coefficient (r) with their corresponding p-value (∗∗∗, p-value <0.001).

To investigate molecular differences among CRC tissue compartments under different preservation methods, we conducted pairwise of DEGs analyses between PanCK+ vs PanCK-/CD45- segment and between CD45+ vs PanCK-/CD45- segments for each tissue type (Fig. 4A). The volcano plots (Fig. 4A–top row) showed that all samples (FFPE, FF and SF) exhibited a higher number of significantly upregulated genes (p-adj <0.05) with larger absolute log_2_ fold changes >2 in PanCK+ vs PanCK-/CD45- comparisons. Meanwhile, only FF sample showed four significant DEGs (p-adj <0.05), *TRBC1*, *ADAMDEC1*, *PTPRC* and *RGS1*, with more modest expression differences (absolute log_2_ fold changes >2), particularly in the CD45+ vs PanCK-/CD45- (Fig. 4A, bottom row).

A Venn diagram illustrated the overlap among the top 300 upregulated DEGs (p-adj <0.05) from the comparison between PanCK+ and PanCK-/CD45- segments across the three tissue preservation types, revealing 132 shared genes (Fig. 4B). Furthermore, we explored the characteristics of the gene counts for 132 intersecting DEGs that differed between tissue types. For this, we applied the pairwise comparison for 132 genes. Fig. 4C displays density plots comparing the distribution of expression levels for the 132 shared DEGs and all expressed genes in PanCK+ and PanCK-/CD45- segments across the different tissue preservation methods. Among comparisons, we found that the distribution count of 132 intersecting DEGs could be of high expression levels in PanCK+ and low expression levels in PanCK-/CD45- segment. A peak in the low expression range indicated that DEGs were less expressed for PanCK-/CD45- in the FFPE vs FF and the FFPE vs SF data. Correlation analysis of the 132 shared DEGs demonstrated strong concordance between sample types, FF vs SF, FFPE vs FF and FFPE vs SF (R = 0.956, 0.940 and 0.891, respectively). This finding supported that the FFPE tissue from CRC is adequate for the spatial whole transcriptome analysis.

### 3.4 Pathway enrichment analysis of the top intersecting genes across tissues

**Fig. 5:**
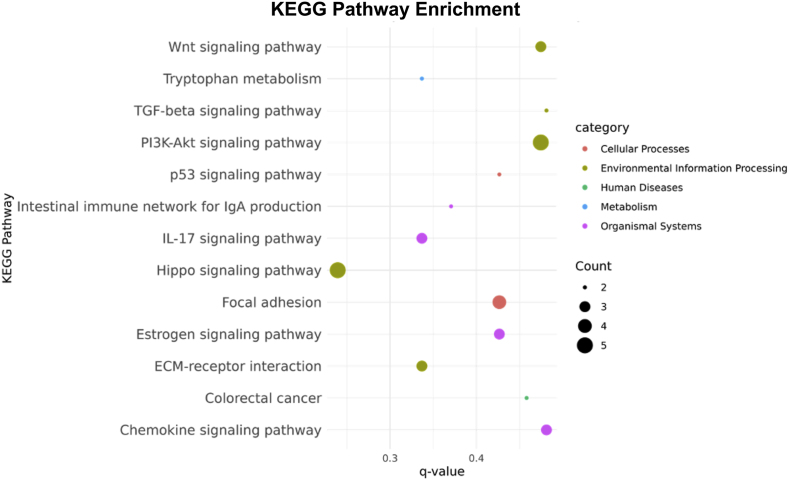
**Pathway enrichment analysis of the top intersecting genes across tissues.** Bubble diagram of the KEGG pathway from the DEG analysis. The pathways were categorized in five groups; cellular process (red), environmental information processing (dark green), human disease (light green), metabolism (blue) and organismal systems (pink). The Benjamini-Hochberg correction was applied which use a significance threshold of p-adj <0.05 (q-value).

To determine critical biological processes and molecular pathways, 132 significant intersecting genes across tissues were imported into enrichment analysis. The results of KEGG pathway enrichment showed that their DEGs were enriched in the associated CRC pathways such as the Wnt signaling, TGF-β, and P53 signaling pathways for all tissue types (Fig. 5).

To identify the unique pathways captured by each preservation method, we performed pathway enrichment analysis on the distinct expressed genes between the PanCK+ and PanCK-/CD45- segments of the FFPE, FF, and SF samples, which consisted of 103, 84, and 85 genes, respectively (Fig. 4B). We found that the uniquely expressed genes in the FF and SF samples were predominantly enriched in cellular metabolism pathways, particularly those related to reactive oxygen species (ROS). For example, terpenoid backbone biosynthesis and pyruvate metabolism were enriched in the FF sample, while chemical carcinogenesis-ROS and oxidative phosphorylation pathways were enriched in the SF samples. In contrast, the FFPE sample showed limited enrichment in ROS-related pathways. These findings may suggest that frozen preservation methods, both FF and SF, better preserve mitochondrial function compared with FFPE (Supplementary Fig. 2). Interestingly, only expressed genes in FFPE were enriched in neutrophil extracellular trap pathway.

## 4 Discussion

To date, spatial whole transcriptomic technologies provide both opportunities and challenges. A fundamental challenge in the accurate analysis of transcriptomic data lies in the quality of tissue samples. It is imperative to assess whether data derived from various tissue types—especially FFPE—are suitable for downstream analysis to ensure the validity of the conclusions drawn. Comparative transcriptomic profiles generally assume that most genes are not differentially expressed across preservation conditions and therefore follow a similar distribution. In this study, we show that all tissue preservation methods from a patient with stage IV CRC provide biological information to study CRC heterogeneity.

The DSP platform allows high-plex morphology-guided analysis within defined regions of FFPE tissue [11]. For comparison of gene expression in three distinct tissue samples, we defined three different segments in each sample. Normalization data demonstrated that FFPE tissue showed the highest average expression level across all genes for CD45+ and PanCK-/CD45- segments, suggesting that using FFPE tissue in spatial approaches will be essential for elucidating the architectural complexity of the TME in CRC tumors. Comparing three different tissue types further revealed intertissue and intratissue heterogeneity for transcriptomic spatial analysis. The frozen tissues showed different data distributions from FFPE tissue. Tissue-type-specific qualities and tissue processes may be correlated with differences in data distribution and low average expression across all genes in the NanoString GeoMx DSP analysis [27,28].

Studying TME in CRC enables the discovery of novel targeted therapies and provides insights into the mechanisms of disease [1,9,10]. FFPE showed significant DEGs between CD45+ and PanCK-/CD45- segments, which represent components of TME. This finding may suggest that cells in the TME compartment had lower expression than cancer cells. We were unable to deconvolute cell types across different segments in each tissue; therefore, further studies are needed to explore this. We applied a gene enrichment analysis for 132 intersecting genes among three different tissue types. The results confirm that all tissue types exhibit biological pathways such as the Wnt, TGF-β, and P53 signaling pathways which are related to the characteristics of CRC.

The accumulation of genetic alterations in driver genes contributes to the initiation and progression of CRC. Mutations in the Wnt, P53, and TGF-β genes have long been implicated in CRC carcinogenesis and progression [3,29]. Alterations in the Wnt and P53 signaling pathways are frequently observed in CRC [3,29]. In addition, TGF-β, signaling is linked to the activation of the epithelial–mesenchymal transition (EMT) pathway and is strongly associated with immune escape within the tumor immune microenvironment [3,10]. While FFPE samples may be generally sufficient for studying spatial gene expression profiles of key and prominent features in CRC, they may not be suitable for detailed analyses of specific pathways or new gene clusters. For such purposes, FF or SF tissues are preferred.

There are several limitations to this study. The most substantial limitation is the sample size. Although four regions were analyzed within each tissue type (36 AOIs), all data were derived from a single biological specimen. This restricts the generalizability of our findings and prevents any conclusions regarding inter-patient variability, CRC subtypes, or stage-specific effects. However, this study was designed as a proof-of-concept feasibility investigation to evaluate the technical comparability of different preservation methods within the same biological background under tightly controlled conditions. All samples were processed within a single batch, eliminating batch effects; nevertheless, investigating batch-to-batch variation would provide additional insight. To strengthen these observations, future studies should include a larger cohort to validate the findings and assess biological variability.

## 5 Conclusion

This study demonstrates that spatial whole transcriptome analysis using the GeoMx DSP platform can be effectively applied to CRC tissues preserved by FFPE, FF, and SF methods. Despite lower fold changes observed in FFPE samples, key transcriptomic signatures were consistently preserved, supporting the reliability of FFPE tissues for spatial transcriptomics. While frozen tissues yielded higher overall gene expression, FFPE samples maintained adequate transcript integrity and spatial resolution, enabling meaningful biological insights. Collectively, our findings suggest FF and SF tissues provide higher gene expression levels, while FFPE tissue preserves low-abundance transcripts that are critical for spatial profiling of CRC.

## Declaration of generative AI and AI-assisted technologies in the manuscript preparation process

The authors confirm that no generative AI or AI-assisted technologies were used in preparing this manuscript.

## Ethical consideration

The study protocol and the informed consent procedures were reviewed and approved by the Siriraj Institutional Review Board (certificates of approval number Si105/2021). All methods were performed in accordance with the relevant guidelines and regulations. A participant was informed about the study through both verbal explanation and written documentation as well as provided signed written informed consent prior to enrollment.

## Funding

This study is supported by Siriraj Research Development Fund (Managed by Routine to Research, R2R): R016635057 and the Foundation for Cancer Care, Siriraj Hospital: R016241047, Thailand. This study is partially supported by the Health Systems Research Institute (HSRI), Thailand (63–117 and 66–083).

## CRediT authorship contribution statement

**Kullanist Thanormjit:** Data curation, Formal analysis, Funding acquisition, Methodology, Visualization, Writing – original draft, Writing – review & editing. **Thanawat Suwatthanarak:** Data curation, Formal analysis, Methodology, Supervision, Writing – review & editing. **Tantip Arigul:** Data curation, Formal analysis, Methodology, Visualization, Writing – review & editing. **Amphun Chaiboonchoe:** Data curation, Formal analysis, Methodology, Writing – review & editing. **Onchira Acharayothin:** Methodology, Writing – review & editing. **Piroon Jenjaroenpun:** Formal analysis, Supervision, Visualization, Writing – review & editing. **Vitoon Chinswangwatanakul:** Funding acquisition, Resources, Supervision, Writing – review & editing. **Pariyada Tanjak:** Conceptualization, Data curation, Formal analysis, Funding acquisition, Methodology, Supervision, Visualization, Writing – original draft, Writing – review & editing.

## Declaration of competing interest

The authors declare the following financial interests/personal relationships which may be considered as potential competing interests:Pariyada Tanjak reports financial support was provided by the Health Systems Research Institute (HSRI), Thailand (63–117 and 66–083). Vitoon Chinswangwatanakul reports financial support was provided by the Foundation for Cancer Care, Siriraj Hospital, Thailand (R016241047). Kullanist Thanormjit reports financial support was provided by Siriraj Research Development Fund (Managed by R2R, R016635057). If there are other authors, they declare that they have no known competing financial interests or personal relationships that could have appeared to influence the work reported in this paper.

## Data Availability

The normalized gene expression data in this study are available in supplementary file 1. The analysis codes in this study are available in supplementary file 2. The research data are provided in supplement figures. The raw sequencing data used in this study are available in the Gene Expression Omnibus (accession number: GSE310215). Other data are available from the corresponding author upon request.
