# Supplementary material for: Comparative spatial whole transcriptome analysis of matched frozen and formalin-fixed paraffin-embedded colorectal cancer tissues
Source: Biochem Biophys Rep. 2025 Dec 16;45:102413. doi: 10.1016/j.bbrep.2025.102413 (PMC12808533; doi:10.1016/j.bbrep.2025.102413)
Supplement: Multimedia component 3 [file mmc3.pdf]

CD45+

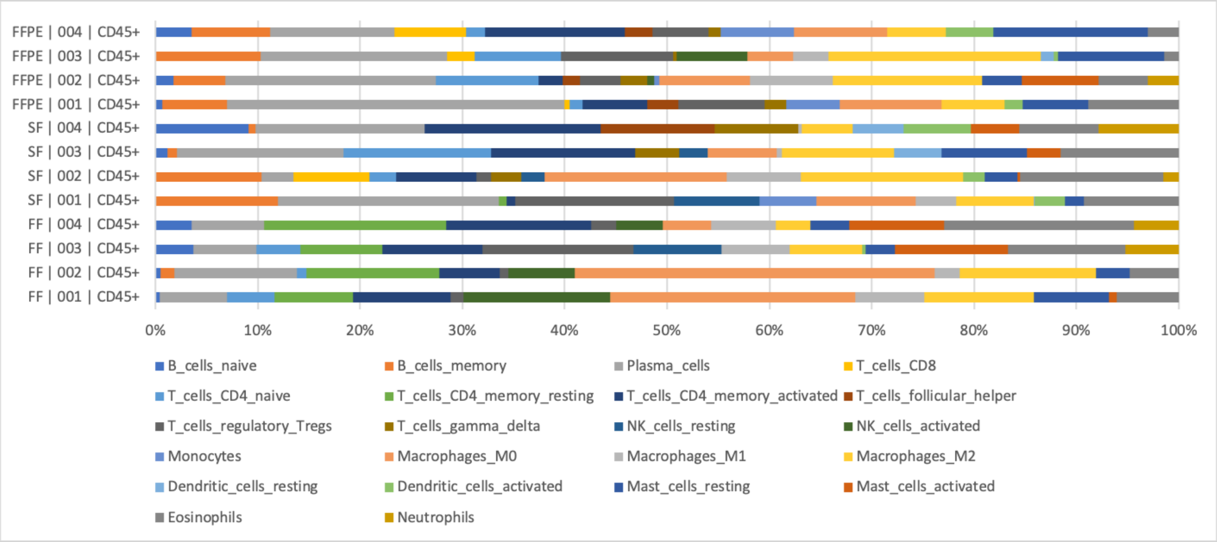

PanCK-/CD45-

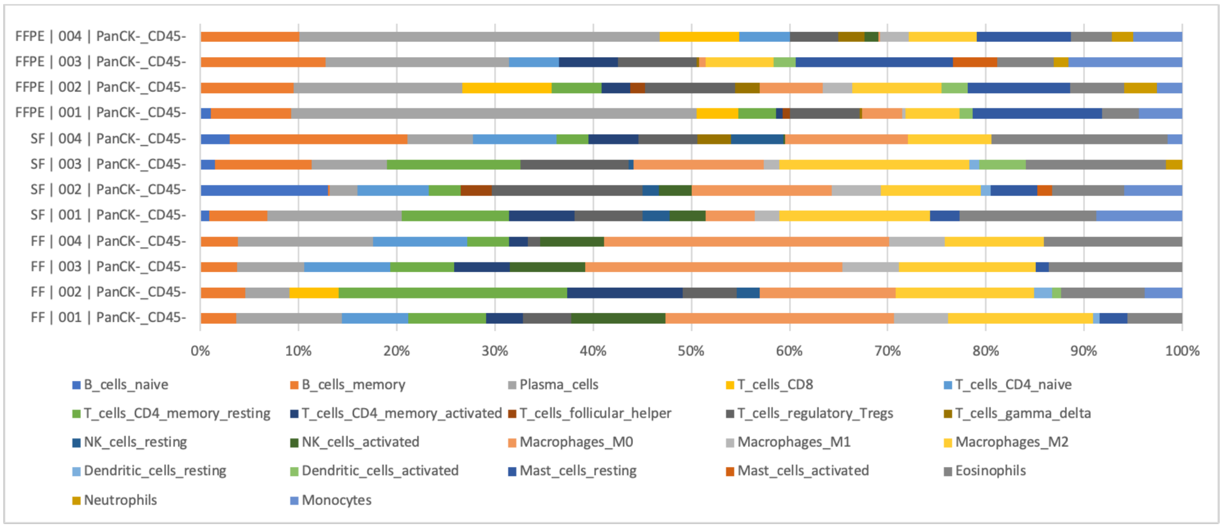

Supplementary figure 1: Immune cell type deconvolution between CD45+ vs PanCK-/CD45- across different preservation methods, FFPE SF and FF. Deconvolution of expression data was executed using GEDIT (The Gene Expression Deconvolution Interactive Tool) [1] using LM22 [2] as a reference matrix, using the default parameters. This approach finds the optimal solution to determine the most likely total cell composition for a particular sample.

References:

1. Nadel, B. B., Lopez, D., Montoya, D. J., Ma, F., Waddel, H., Khan, M. M., Mangul, S., & Pellegrini, M. (2021). The Gene Expression Deconvolution Interactive Tool (GEDIT): accurate cell type quantification from gene expression data. GigaScience, 10(2), giab002. https://doi.org/10.1093/gigascience/giab002

2. Newman, A. M., Liu, C. L., Green, M. R., Gentles, A. J., Feng, W., Xu, Y., Hoang, C. D., Diehn, M., & Alizadeh, A. A. (2015). Robust enumeration of cell subsets from tissue expression profiles. Nature methods, 12(5), 453–457. https://doi.org/10.1038/nmeth.3337
