# Supplementary material for: Comparative spatial whole transcriptome analysis of matched frozen and formalin-fixed paraffin-embedded colorectal cancer tissues
Source: Biochem Biophys Rep. 2025 Dec 16;45:102413. doi: 10.1016/j.bbrep.2025.102413 (PMC12808533; doi:10.1016/j.bbrep.2025.102413)
Supplement: Multimedia component 4 [file mmc4.pdf]

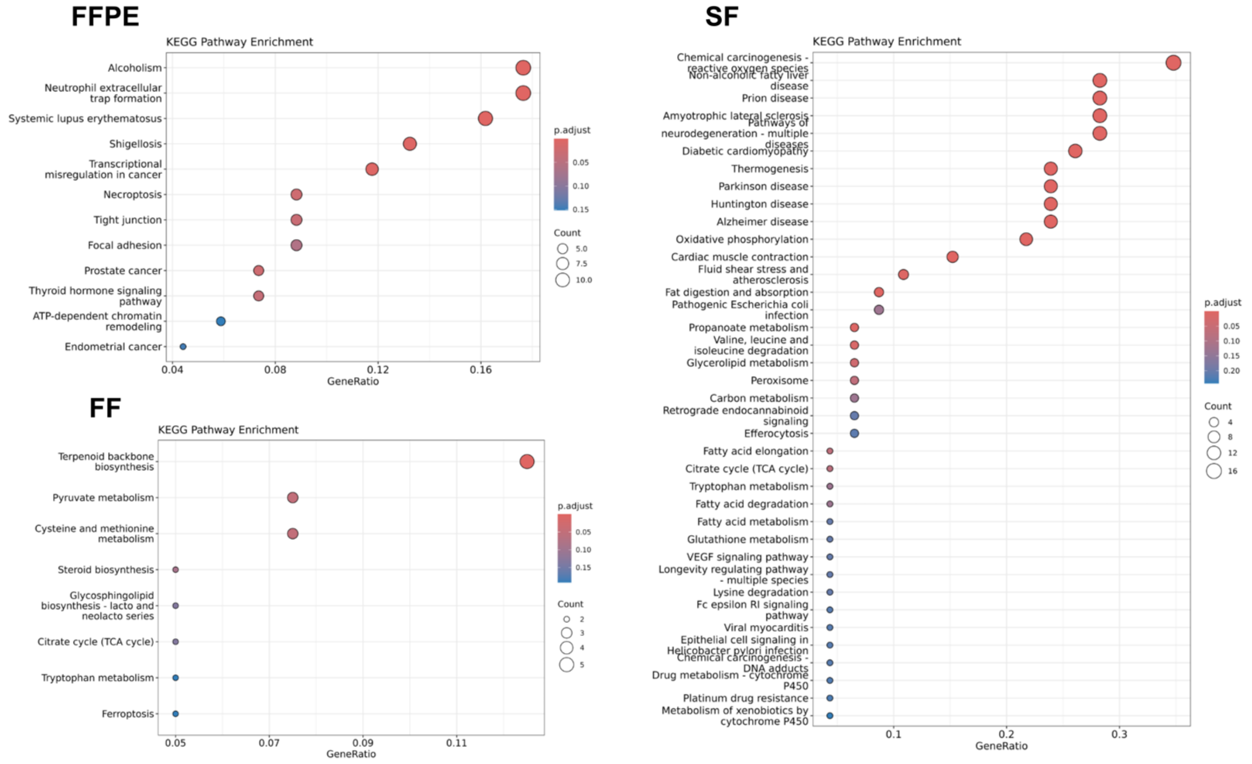

Supplementary figure 2: Kyoto Encyclopedia of Genes and Genomes (KEGG) pathway enrichment analysis in each preservation method.
